# A novel approach to increasing community capacity for weight management a volunteer-delivered programme (ActWELL) initiated within breast screening clinics: a randomised controlled trial

**DOI:** 10.1186/s12966-021-01099-7

**Published:** 2021-03-06

**Authors:** Annie S. Anderson, Huey Yi Chong, Angela M. Craigie, Peter T. Donnan, Stephanie Gallant, Amy Hickman, Chloe McAdam, Jennifer McKell, Paul McNamee, E. Jane Macaskill, Nanette Mutrie, Ronan E. O’Carroll, Petra Rauchhaus, Naveed Sattar, Martine Stead, Shaun Treweek

**Affiliations:** 1 Centre for Research into Cancer Prevention and Screening, University of Dundee, Ninewells Hospital & Medical School, Dundee, DD1 9SY UK; 2 Health Economics Research Unit, Institute of Applied Health Sciences, University of Aberdeen, Aberdeen, AB25 2ZD UK; 3 Division of Population Health and Genomics, University of Dundee, Ninewells Hospital & Medical School, Dundee, DD1 9SY UK; 4 Breast Cancer Now, 222 Leith Walk, Edinburgh, EH6 5EQ UK; 5 Physical Activity for Health Research Centre, University of Edinburgh, Saint Leonard’s Land, Holyrood Rd, Edinburgh, EH8 8AQ UK; 6 Institute for Social Marketing and Health, Faculty of Health Sciences and Sport, University of Stirling, Stirling, FK9 4LA UK; 7 Department of Breast Surgery, Level 6, Ninewells Hospital and Medical School, Dundee, DD1 9SY UK; 8 University of Stirling, Stirling, FK9 4LA UK; 9 Tayside Clinical Trials Unit, Tayside Medical Science Centre, Ninewells Hospital and Medical School, Dundee, DD1 9SY UK; 10 University of Glasgow, Institute of Cardiovascular and Medical Sciences, BHF Glasgow Cardiovascular Research Centre, 126 University Place, Glasgow, G12 8TA UK; 11 Health Services Research Unit, University of Aberdeen, , Room 306, 3rd Floor, Health Sciences Building, Foresterhill, Aberdeen, AB25 2ZD UK

**Keywords:** Breast cancer body weight lifestyle, Intervention screening, Physical activity, Cost-effectiveness analysis

## Abstract

**Background:**

It is estimated that around 30% of breast cancers in post-menopausal women are related to lifestyle. The breast cancer-pooling project demonstrated that sustained weight loss of 2 to 4.5 kg is associated with an 18% lower risk of breast cancer, highlighting the importance of small changes in body weight. Our study aimed to assess the effectiveness a volunteer-delivered, community based, weight management programme (ActWELL) for women with a BMI > 25 kg/m^2^ attending NHS Scotland Breast Screening clinics.

**Methods:**

A multicentre, 1:1 parallel group, randomised controlled trial was undertaken in 560 women aged 50 to 70 years with BMI > 25 kg/m^2^. On completion of baseline measures, all participants received a breast cancer prevention leaflet. Intervention group participants received the ActWELL intervention which focussed on personalised diet advice and pedometer walking plans. The programme was delivered in leisure centres by (the charity) *Breast Cancer Now* volunteer coaches.

Primary outcomes were changes between groups at 12 months in body weight (kg) and physical activity (accelerometer measured step count).

**Results:**

Two hundred seventy-nine women were allocated to the intervention group and 281 to the comparison group. Twelve-month data were available from 240 (81%) intervention and 227 (85%) comparison group participants. Coaches delivered 523 coaching sessions and 1915 support calls to 279 intervention participants. Mean weight change was − 2.5 kg (95% CI − 3.1 to − 1.9) in the intervention group and − 1.2 kg (− 1.8 to 0.6) in the comparison group. The adjusted mean difference was − 1.3 kg (95% CI − 2.2 to − 0.4, *P* = 0.003). The odds ratio for losing 5% weight was 2.20 (95% CI 1.4 to 3.4, *p* = 0.0005) in favour of the intervention. The adjusted mean difference in step counts between groups was 483 steps/day (95% CI − 635 to 1602) (NS).

**Conclusions:**

A community weight management intervention initiated at breast screening clinics and delivered by volunteer coaches doubled the likelihood of clinically significant weight loss at 12 months (compared with usual care) offering significant potential to decrease breast cancer risk.

**Trial registration:**

Database of registration: ISCRTN.

Registration number:11057518.

Date trial registered:21.07.2017.

Date of enrolment of first participant: 01.09.2017.

**Supplementary Information:**

The online version contains supplementary material available at 10.1186/s12966-021-01099-7.

## Introduction

Breast cancer is the most common cancer in women worldwide. In Scotland, the disease accounts for 29% of all cancers diagnosed [1]. Incidence is increasing and current predictions from ISD (Scottish Government) suggest a rise of 27.5% between 2008 and 2012 and 2023–2027 [2].

Whilst many factors are implicated in the aetiology, current estimates suggest that around 30% of breast cancers in post-menopausal women are related to physical inactivity, alcohol consumption and body fatness [3]. Gaining weight in adult life is also a strong predictor of breast cancer (especially in women who have not taken hormone replacement therapy) [4]. The incidence of breast cancer differs from other obesity related cancers in that rates are 14% lower for women living in the most deprived areas compared with the least deprived [5].

Observational data has consistently shown that relatively modest weight losses in post-menopausal women are associated with significant risk reduction in breast cancer [4, 6, 7]. A report from the breast cancer-pooling project demonstrated that modest, sustained intentional weight loss (over 10 years) of 2 to 4.5 kg is associated with an 18% lower risk of the disease [8]. This magnitude of change is similar to the 3% body weight loss noted by NICE as associated with reductions in obesity related co-morbidities [9].

Women meeting at least five of the World Cancer Research Fund prevention guidelines for lifestyle (including physical activity and alcohol) show a 60% lower risk for breast cancer compared to women meeting none of the guidelines [10]. A recent systematic review reported that high versus low adherence to cancer prevention guidelines was associated with consistent reductions in breast cancer incidence [11]. Whilst awareness about obesity related behaviours and other lifestyle habits and cancer risk is increasing [12] there are few interventions (policy or individual level) directed towards supporting weight management and other modifiable breast cancers risk factors in women approaching or after menopause [13].

Most (71%) Scottish women aged 50 to 70 years accept invitations to attend NHS Scotland Breast Screening Programme (NHSSBSP) every 3 years and as such the service provides a unique opportunity to endorse and support lifestyle interventions for large numbers of women [14]. The cancer research “gap analysis” reviews by Breast Cancer Campaign [4, 15] highlighted the role of breast screening programmes as an opportunistic “teachable moment” for promoting cancer prevention activities.

Actions at policy level potentially offer equitable approaches to assist the population to achieve desirable changes in weight-related behaviours but in the UK these are limited and principally focussed on childhood obesity. In Scotland, the breast cancer community is currently exploring innovative and sustainable preventative opportunities to support weight management programmes for breast cancer risk reduction utilising existing community asset approaches including volunteers, physical resources such as leisure centres and existing NHS screening services.

We aimed to evaluate the impact of a novel community based, weight management programme (ActWELL) in women with a body mass index (BMI) > 25 kg/m^2^ attending routine NHS breast screening clinics.

## Methods

The study design was a multicentre, 1:1 parallel group, randomised controlled trial conducted in four NHSSBSP screening centres in Scotland from August 2017 to September 2019. The full protocol is reported elsewhere [16]. See also CONSORT checklist.

### Sample size

Using the data from women with excess body weight (BMI > 25 kg/m^2^) in the ActWELL feasibility study [17] (mean body weight 80.9 ± 17.9 kg), a total of 414 women (207 per group) were required to detect a 7% weight between groups change at 90% power (alpha 0.05). Allowing for at a 25% drop out (based on our findings from the feasibility study) we sought to randomise a sample size of 552. The feasibility study randomised 10 women per site per month and we estimated that it would take up to 14 months for four sites to attain the sample size in this trial (allowing for holidays).

Based on the ‘Walking for Wellbeing in the West’ study [18] we estimated that to detect a difference of 2000 steps between groups at follow-up, at 90% power, 102 women (51 per group) would be required to provide data at both time points. Allowing for 20% drop out (plus any equipment malfunction/postal losses) we aimed to recruit a further 30% bringing the total enrolment to 146 of the 552 participants for this aspect of the study.

Our sample size was based on numbers of women needed to provide meaningful data on both primary outcomes recognising that much smaller numbers were needed for assessing changes in physical activity.

### Participants and recruitment

We requested that all attendees at routine NHS breast screening appointments were informed about the study by (NHSSBSP) staff via a leaflet (given by clinic reception staff at check in) and a brief 30-s introductory conversation verbally after mammographic procedures were completed. Interested clients were asked to complete an invitation card and leave it in a dedicated box at the appointment venue. Respondents were then contacted by research nurses to assess BMI and other eligibility criteria.

#### Inclusion Criteria

- Attending, or invited to attend, routine breast screening clinics (not recall clinics)
- Measured BMI > 25 kg/m^2^
- Age 50 to 70 years

#### Exclusion Criteria

- Currently undergoing treatment for any malignant condition (excluding basal or squamous cell skin cancers)
- Reported contra-indication to physical activity (e.g. recent surgery)
- Reported contra-indication to weight loss (e.g. currently following a recovery programme for weight gain)
- On a specialised medical diet e.g. gluten free
- Diagnosis of Type 1 diabetes
- Current use of insulin
- No telephone contact
- Unable to consent

Respondents were contacted by telephone on a “first come, first served” basis until all the available research nurse appointments for the week were filled. Women who were not contacted about the study were sent information on reducing breast cancer risk as were women who were contacted but found to be ineligible for the study. No cards were held for future contact. The number of appointments (e.g. staff time made available) was based on the results of the feasibility study where 10 participants per week per clinic were successfully recruited. Participants who are considered eligible were invited to attend their local research centre to provide informed consent before commencing baseline measures.

During preparation for recruitment, senior screening management staff reported that they a) were reluctant or unable to provide data on number of attendances, and the numbers of cards given out, b) staff who did not feel comfortable introducing the concept of lifestyle would not be compelled to do so, c) verbal information on the study would only be provided if time permitted (and may be difficult in busy clinics), d) communications on mobile screening units were considered difficult at some sites because no reception staff were available. It also became apparent that the demographic backgrounds of screening attendees were dependent on which general practice areas were being invited for screening and where mobile screening vans were sent.

### Assessment measures

Procedures are outlined here and full details are provided elsewhere [16]. A full list of measured outcomes is presented in Table 1. Research nurses at each site collected data at baseline, 12 weeks (by telephone) and 12 months. At baseline, demographic data were collected.

**Table 1:** Assessment measures

OUTCOME MEASURES	Base line	12 week	12 month
**Primary Outcomes**			
Body weight			
Measured using digital body weight scales (kg)	X		X
Physical Activity			
7 days accelerometry with activPAL^TM^ (steps) (subsample only)	X		X
**Secondary outcomes**			
Modes of physical activity Sedentary behaviour			
Scottish Physical Activity Questionnaire SPAQ [19]	X	X	X
7 days accelerometry with activPAL™ (subsample only)	X		X
Anthropometric changes			
BMI (height and weight) Waist circumference (cm)	X		X
Eating habits			
Questionnaire based on Scottish Health Survey [20]	X		X
Fruit and vegetable intake [21]		X	
Alcohol intake			
Audit C questionnaire [22]	X		X
Psycho-social variables			
Modified brief illness questionnaire [23]	X		X
Knowledge and beliefs about lifestyle and breast cancer risk (developed in house)	X		X
Psychosocial health measures resources (perceived motivation, awareness, ability, action, monitoring, and social support around weight management) (developed in house)			X
Perceived body weight	X	X	X
Economic outcomes			
EQ 5D-5L questionnaire [24]	X	X	X
Economic health resource usage (Developed by HERU, University of Aberdeen)	X		X

The two primary outcomes were measured change in body weight and change in step count (physical activity) by 12 months. Both were measured as the mean difference between groups at 12 months adjusted for baseline weight, site, minimisation variables, Scottish Index of Multiple Deprivation (SIMD) [25], ethnicity, home ownership and number of coach sessions. Physical activity was objectively measured using thigh worn activPAL™ (PAL Technologies Ltd., Glasgow, UK) accelerometers. A minimum of 4 days from a participant were required for the activPAL™ data to be considered valid [26].

Secondary outcomes were measured changes in waist circumference, self-reported moderate/vigorous minutes of physical activity (work, leisure, housework), sedentary behaviour (sitting time), eating habits, alcohol intake, psychosocial variables, HbA1C, CVD risk factors (non-fasting lipids) and non-fasting insulin, blood pressure, quality of life and health economic outcomes. Details of measurement tools are presented elsewhere [16].

Delivery and Process Outcomes: The lifestyle coaches kept records on intervention delivery and after 5 completed participant sessions reported adherence to key delivery components (scaled as never to always). In addition, audio-recorded coach sessions were assessed for adherence to protocol.

Perceptions, experiences and perceived acceptability of the intervention were attained through qualitative semi-structured interviews. These were conducted with one member of NHSSBSP staff at each site (who had volunteered as an ActWELL champion), volunteer coaches (by site), leisure centre staff (one from each site) and intervention and comparison group participants (selected by site and socio-economic area of residence). All interviews were audio-recorded, transcribed and coded for thematic analysis.

In addition, intervention participants were invited to return exit questionnaires on perceived value of specific components of the intervention programme, acceptability and recommendations to others.

### Randomisation

Following the baseline visit, eligible women who had consented to participate were randomised centrally at a 1 to 1 ratio to receive either the ActWELL programme or usual care (comparison group) using the web-based TRuST system developed and managed by the Tayside Clinical Trials Unit. Randomisation was stratified by site and minimised by socio-economic status based on Social index of Multiple Deprivation (SIMD). In addition, 146 women (73 from each group) were randomly allocated by the TRuST system to receive the activPAL™ monitor (accelerometer) (see ‘Sample size’ above). This second randomisation was done at the baseline visit so that the research nurse could provide and fit the activPAL™ monitor to those allocated to receive it. Participants, coaches, trial manager and administrator were aware of intervention group allocation, but research nurses (who collected data) and the trial statistician were not. It should be noted that both primary outcomes used objective measurement approaches.

### Intervention group

The intervention was based on the COM-B model of behaviour change [27]. This incorporated increased capability for lifestyle change (via a volunteer coach delivered personalised programme), enhanced opportunities for greater physical activity (via pedometer-based programmes and introduction to local leisure centres) and increased motivation for weight management e.g. by raising awareness of breast cancer risk reduction within screening.

The programme was delivered in two individual, one to one sessions (60 min and 45 min) in the first 12 weeks of the intervention period and 9 (15-min) support calls over the following 9 months, totalling 4 h contact over a 12-month period. The programme was delivered by volunteer coaches who were recruited and managed by the charity Breast Cancer Now. The charity recruited volunteers who had relevant experience with assisting people undertake life changes (e.g. nurses, teachers, church work) and they underwent a 2 day bespoke training programme from the experts in the research team (including physical activity and dietetics). Coaches were then asked to undertake 2 full coaching sessions (with feedback) from participant volunteers. On going support (e.g. frequently asked questions, local WhatsApp group for coaches and questions and queries were handled by the Breast Cancer Now project officer on an on-going basis.

Face-to-face visits between volunteer coach and participants took place in non-gym space (e.g. an office) in local leisure centres. The main physical activity component of the intervention was a pedometer-based walking programme, introduced at the first face-to-face visit with a 10-min “walk and talk” session. Participants were supported to increase physical activity towards accumulating at least 150 min of moderate intensity physical activity per week through the provision of graduated walking goals and then, where appropriate, towards 300 min per week (Based on Scottish Intercollegiate Guidelines Network (SIGN)  [28] guidance on weight management).

All intervention participants were set a target goal of a 7% reduction in body weight and provided with a personalised energy prescription of 2508 kJ (600 kcal) below that required for weight maintenance. Bodyweight scales were offered in order to undertake self-monitoring. If the weight loss target was attained then guidance was given on weight loss maintenance. Behavioural change techniques (BCTs) included education, motivational interviewing, goal setting, action and coping planning implementation intentions, self-monitoring of body weight and steps and feedback. The content and design of the programme was based on the feasibility study findings, views of the target group and those involved in facilitating the programme. At the end of the study we also offered referrals to NHS weight loss services to women who still had a BMI > 25 kg/m2 as well as information on other weight management programmes (including internet based programmes). Full details of programme optimisation are described elsewhere [29]. The details of the programme content are outlined in Table 2.

**Table 2:** Key components of the lifestyle coach sessions (face to face visits)

Visit 1 – Face-to-face (60 min)	Visit 2 – Face-to-face (45 min)
• identification of BMI • Instruct participant on pedometer use and proposed walking programme • Walk and talk 10 min (interactive walking session) • Physical activity goal setting (implementation intention setting and personalised walking programme) • Discuss how to reduce sedentary behaviour • Caloric value of (hot and cold) alcohol and sugary drinks discussed “Sugar Savvy” quiz undertaken (https://www.wcrf-uk.org/sites/default/files/are-yousugar-savvy-game.pdf) (advice given on alternatives, portion size, frequency) (Possibility of implementation intention setting on drinks) • Weight loss goal (emphasis on modest up to 7% in 12 months) • Motivational interviewing questions on weight loss • Guidance on self-monitoring, weekly self-weighing, reporting and feedback– implementation intention setting for weighing • Initial dietary challenges – snacking and “weakness foods” (based on a verbal 24 h intake) • Summarise meeting – goal setting, action and coping planning, times of relapse	• Praise success (however modest) • Evaluate and modify PA goals as required. Check body weight recorded • Reminder about body weight and breast cancer risk reduction (even after 50) • Highlight weight loss principles (revising snacking, importance of meal patterns and 5 a day) • Remind about goal set for weight loss and how this converts to personal eating plan • Review 24 h diet recall sheets (handed out last visit) (or take a 24 h recall if sheets not completed) • Discuss calories – focus on -600 kcal deficit diet (Identify personalised eating plan using British Heart Foundation (BHF) materials) • Discuss Portion sizes and frequencies (use images from BHF materials and portion distortion information) • Food labelling • Identify Implementation intentions on one food/drinking habit (set one only- if suggestions needed base on 24 h recordings) • Summarise goals and key challenges, check all materials provided • Arrange first two telephone appointments • Discuss leisure centre activity to meet staff (if interested)
**Nine, follow-up phone calls**	
• Check well being	
• Check goal progress, self-reported weight, re-enforce the importance of self-monitoring	
• Identify success and challenges	
• Discuss possible problems ahead (e.g. holidays)	
• Coping strategies and starting again if intentions failed	
• Start discussion on the importance of habits in eating behaviours using Ten Top Tips.	
• Weight Loss and Weight Loss Maintenance	
• Highlight the importance of regular food intake (including breakfast) and portion size Refer to Keep to your meal routine and Focus on Food	
• Stress the importance of physical activity and social support Refer to Tips Walk off the weight	
• Re-enforce importance of self-monitoring	
• Re-enforce information on snacking Refer to Pack a Healthy snack and Five a Day	
• Re-enforce information on drinks sweet and alcohol and value of water Refer to Think about your drinks	
• Re-evaluate portions size (as per BHF booklet) Refer to Caution with your portions	
• Return to discussing physical activity and reducing sedentary behaviour Refer to Up on your feet	
• Re-evaluation of goals, coping planning, where next, summarise success	

### Comparison group

All participants (including all the comparison group participants) underwent all data collection procedures at baseline, 12 weeks and 12 months including weighing. On completion of baseline measures all participants received a breast cancer prevention leaflet [30] (which noted the relevance of lifestyle factors). On completion of their 12 months follow up visit women in the comparison group were offered a single personalised coaching session from volunteers and the ActWELL intervention written pack.

### Primary and secondary outcomes

#### Analysis

Full details of analytical procedures are described elsewhere and based on a pre-datalock Statistical Analysis Plan (SAP) [16]. In summary, the primary analysis used an intention to treat analysis with all available data using mixed models. We carried out multiple linear regression analyses with mixed effects models adjusted for the corresponding baseline values with group allocation, minimisation variables SIMD, site, ethnicity, home ownership and number of coach sessions as fixed effects. Sensitivity analysis utilised measures of Akaike’s Information Criterion (AIC) to estimate the fit of models. Transformations were used for secondary outcomes where normality was not plausible. SAS software (version 9.4, SAS Institute Inc., Cary, NC, USA) was used for all statistical analyses.

The within-trial health economics analysis adopted the perspective of the UK NHS and compared the health care costs and health-related quality of life effects (measured by the ED-5D-5L instrument, summarised by changes in Quality Adjusted Life Years, “QALYs”) for both groups over 12 months after randomisation. Costs are reported in 2017/18 pounds sterling (£), using NHS reference cost 2017/18 and the Unit Costs of Health & Social Care 2018 to value the resource use [31, 32] with adjustments made for inflation using the Hospital & Community Health Services (HCHS) Index and the new Health Services Index using CPI (Health).

Self-reported health care resource use measurement included costs in primary care (general practitioner, district nurse, practice nurse and other community health professional visits) and secondary care (emergency, hospital admissions and hospital outpatient attendances). ActWELL intervention costs comprised the Breast Cancer Now Co-ordinator salary, (adjusted to reflect only time spent on intervention delivery activities, such as recruiting and co-ordinating volunteers), management cost of Breast Cancer Now, training time and associated expenditure, consumables (training packs and participant materials) and travel costs for volunteer coaches to reach training centres, update visits and leisure centres.

#### Patient and public involvement

The intervention development (invitation design and wording, content, pitch, interactive materials, use of behaviour change techniques) at feasibility stage and the definitive trial were informed by focus group discussions with women aged 50 to 70 years who had previously received an invitation to attend breast screening. In addition, feedback from participants of the feasibility trial had a direct influence on delivery design (timing, content) for the RCT.

## Results

### Recruitment and follow-up

**Fig. 1:**
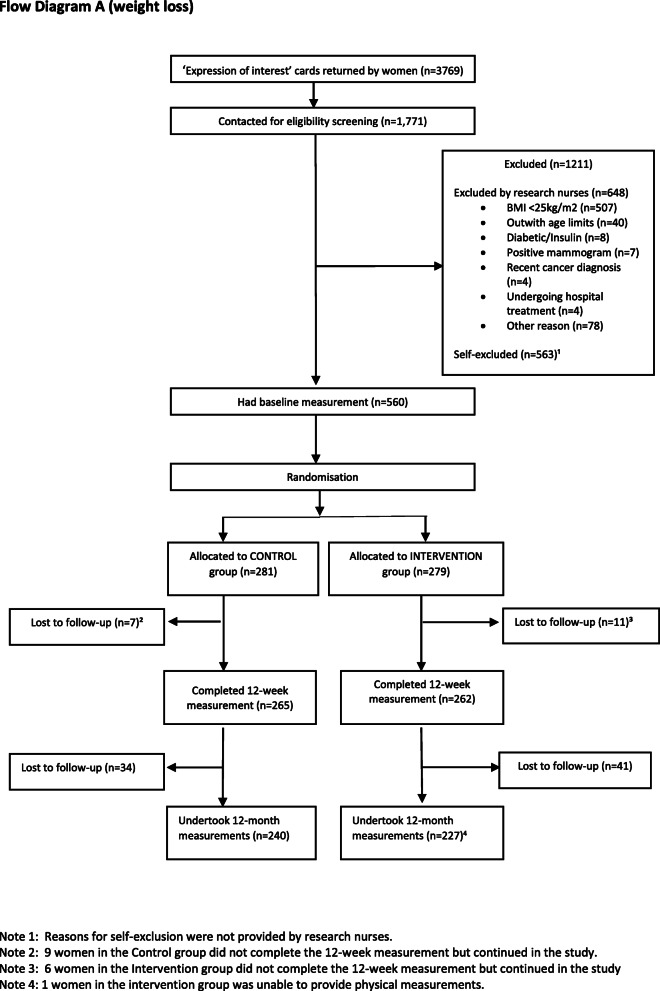
Flow Diagram A (weight loss)

Interest in participating in the ActWELL programme was high, with 3769 women requesting project information over a 12-month period, and recruitment closed ahead of schedule. A total of 1711 respondents were contacted by research nurses for further details. Of these, 507 (28%) were known to have a BMI < 25 kg/m^2^ and therefore ineligible for study inclusion. No information on physical activity was collected at this stage. Around one third (*n* = 563) declined to participate after discussing study requirements and did so without giving reasons [Flow Diagram A] (Fig. 1).

**Fig. 2:**
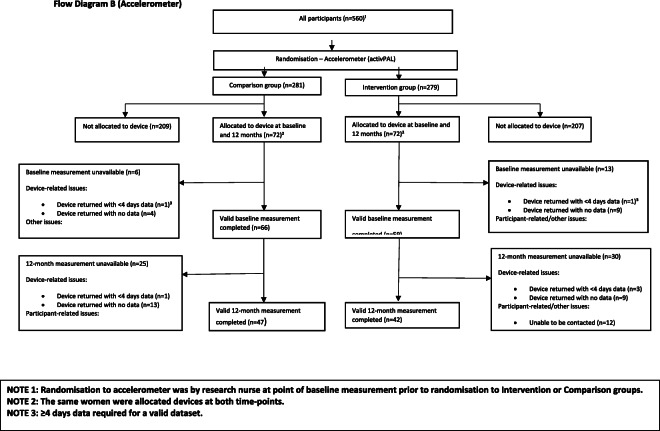
Flow Diagram B (Accelerometer) *(Continued)*

In total, 560 women were randomised (279 to intervention, 281 to comparison). At 12 weeks, 527 (93% intervention, 95% comparison) remained in the study, and at 12 months, 465 (81% intervention, 85% comparison) returned for follow up measures of the primary outcome of body weight [Flow Diagram A] (Fig. 1). In accordance with study size requirements, 144 participants were randomised (72 intervention and 72 comparison) to the activPAL™ accelerometer (second primary outcome) and 125 (93% intervention, 95% comparison) provided at least 4 days of data recording (mean 6.8 days SD 0.74). At 12 months, 89 provided useable data (63% intervention, 67% comparison) [Flow Diagram B] and 82 people provided useable data at both time points (Fig. 2). Device issues and failure to return the activPAL™ units were the major causes for the low number of usable datasets.

### Baseline characteristics

Participants came from all socioeconomic groups; 16% were from SIMD 1 and 2 (highest areas of social deprivation). The mean age at baseline was 59.1 years (SD 5.44) and the majority were well educated and in paid employment (Table 3). Most participants (63.2%) reported being post-menopausal with a further 8.2% as peri-menopausal. Almost half (48.8%) of the randomised participants had a body mass index in the obese category (> 30 kg/m^2^).

**Table 3:** Participant characteristics

	Intervention *n* = 279	Comparison *n* = 281	Total: *n* = 560
Demographic characteristics			
Age (years)			
Mean (SD)	58.8 (5.2)	59.5 (5.7)	59.1 (5.4)
SIMD quintile (%)			
1 (most deprived)	21 (7.5)	15 (5.3)	36 (6.4)
2	25 (9.0)	29 (10.3)	54 (9.6)
3	38 (13.6)	39 (13.9)	77 (13.8)
4	65 (23.3)	60 (21.4)	125 (22.3)
5 (least deprived)	128 (45.9)	135 (48.0)	263 (47.0)
Unknown	2 (0.7)	3 (1.1)	5 (0.9)
Ethnicity (%)			
***White, British***	***265 (95.0%)***	***265 (94.3%)***	***547 (94.6%)***
***White, Irish***	***4 (1.4%)***	***1 (0.4%)***	***5 (0.9%)***
***White, other***	***4 (1.4%)***	***8 (2.8%)***	***12 (2.1%)***
***Mixed***	***0 (0%)***	***2 (0.7%)***	***2 (0.4%)***
***Asian Indian***	***1 (0.4%)***	***1 (0.4%)***	***2 (0.4%)***
***Asian, Pakistani***	***2 (0.7%)***	***0 (0%)***	***2 (0.4%)***
***Asian, Chinese***	***1 (0.4%)***	***0 (0%)***	***1 (0.2%)***
***Asian, other***	***2 (0.7%)***	***1 (0.4%)***	***3 (0.5%)***
***Other***	***0 (0%)***	***2 (0.7%)***	***2 (0.4%)***
Do not wish to complete	0 (0%)	1 (0.4%)	1 (0.2%)
Education, highest level (%)			
Secondary	57 (20.4)	62 (22.1)	119 (21.3)
Other professional/technical qualification	90 (32.3)	84 (29.9)	174 (31.1)
University degree	132 (47.3)	135 (48.0)	267 (47.7)
Employment (%)			
Retired	90 (32.3)	98 (34.9)	188 (33.6)
Unemployed	2 (0.7)	7 (2.5)	9 (1.6)
Employed full-time	91 (32.6)	90 (32.0)	181 (32.3)
Employed part-time	71 (25.4)	65 (23.1)	136 (24.3)
Student full-time	2 (0.7)	0 (0.0)	2 (0.4)
Student part-time	0 (0.0)	1 (0.4)	1 (0.2)
Other (please specify)	23 (8.2)	20 (7.1)	43 (7.7)
Home status (%)			
Owner occupied	255 (91.4)	257 (91.5)	512 (91.4)
Rented	24 (8.6)	24 (8.5)	48 (8.6)

### Outcomes- body weight change

The mean (measured) weight loss at 12 months in the intervention group was 2.5 kg (95% confidence interval (CI) = loss of between 1.9 kg and 3.1 kg) and the weight loss in the comparison group was 1.2 kg (95% CI = loss of between 0.6 kg and 1.8 kg). The primary analysis of weight loss at 12 months adjusted for baseline, site, SIMD and other SAP variables showed a loss of 1.3 kg in favour of the intervention (95% CI = loss of between 0.4 kg and 2.2 kg; *P* = 0.003). Differences in the self-reported body weight between baseline and 12 months also show a significantly greater weight loss in favour of the intervention group after adjustment for all SAP variables (weight loss of 1.2 kg; 95% CI = loss of between 0.3 kg and 2.2 kg; *p* = 0.014). It is notable that self-reported weight continued to decline between 12 weeks and 12 months when the intervention contact was telephone based (Table 4).

**Table 4:** Changes in Body Weight and associated variables

	Intervention Group			Comparison group			Between group difference; *P* value			
	N	Mean (SD)	95% CI	N	Mean (SD)	95% CI	Unadjusted mean (95% CI)	***P*** value	Adjusted for all SAP variables (95% CI)	***P*** value
Changes in anthropometric measures										
Measured body weight (kg)										
Baseline	278	80.9 (13.3)		281	81.9 (12.8)					
12 months	226	77.8 (12.6)		240	80.2 (12.7)					
Difference to baseline	225	−2.5 (4.4)	−3.1 to −1.9	240	−1.2 (5.0)	−1.8 to −0.6	−1.59 (−3.17 to − 0.01)	0.048	−1.29 (−2.15 to − 0.43)	0.003
Self-reported body weight (kg)										
Baseline	267	79.4 (12.9)		272	80.4 (12.7)					
12 weeks	253	78.0 (12.4)		228	79.9 (13.5)					
Difference to baseline	245	−1.5 (3.3)	−1.9 to − 1.1	224	−0.7 (3.4)	− 1.2 to − 0.3				
12 months	219	76.9 (12.7)		224	78.9 (12.7)					
Difference to baseline	210	−2.1 (4.8)	−2.8 to −1.5	219	−0.9 (5.5)	−1.6 to − 0.1	−1.37 (− 2.97 to 0.23)	0.092	−1.23 (− 2.20 to − 0.25)	0.014
Mean waist circumference (cm)										
Baseline	279	98.1 (12.5)		281	98.7 (11.7)					
12 months	226	95.5 (11.7)		239	97.4 (12.0)					
Difference to baseline	226	−2.3 (6.0)	−3.1 to −1.5	239	−1.0 (6.6)	−1.8 to −0.2	− 1.20 (− 2.67 to 0.28)	0.110	−1.24 (− 2.38 to − 0.10)	0.033
BMI (measured)^a^										
Baseline	279	31.0 (4.7)		281	31.3 (4.3)					
12 months	226	29.9 (4.6)		240	30.6 (4.3)					
Difference to baseline	226	−1.0 (1.6)	−1.2 to −0.7	240	−0.5 (1.9)	−0.7 to − 0.2	0.98 (0.97 to 0.10)	0.048	0.98 (0.97 to 0.99)	0.002
Percent weight loss at 12 months^b^										
≥ 5% n (%)	279	76 (27.2%)		281	46 (16.4%)		Odds ratio 2.15 (1.41 to 3.29)	< 0.001	Odds ratio 2.20 (1.41 to 3.43)	< 0.001

^a^log transformed (data presented as back transformations) ^b^ logistic regression allowing for close co-linearity (binary variables)

The mean percentage weight loss (using measured body weight) at 12 months in the intervention group was − 3.07% (95% CI = loss of between 3.77 to 2.37%) compared with − 1.34% (SD 5.76) (95%CI loss of between − 2.07% to − 0.60%) in the comparison group. A greater proportion of intervention participants compared to comparison group attained 5% weight loss. In addition, 51% achieved greater than 2 kg weight loss (the minimum associated with breast cancer risk reduction) (8) in the intervention group compared to 27% in comparison group. It is notable that 19% of women in the intervention group achieved 7% weight loss and were given advice on weight loss maintenance, which constrained the potential of total weight loss in this group.

The mean difference in waist circumference and BMI reductions were significantly greater in the intervention group (Table 4).

#### Outcomes – physical activity change

The mean daily step count at 12 months was lower in both groups than it was at baseline. For those providing valid data (minimum of 4 days of data), in the intervention group the reduction was 69 steps (95% CI − 952 to 1091) and a reduction of 435 steps (95% CI − 1074 to 205) in the comparison group. The primary outcome analysis of adjusted mean difference between groups at 12 months was an additional 483 steps, but this difference was not statistical significant from baseline (Table 5).

**Table 5:** Changes in physical activity measures

Baseline and follow up measures										
	Intervention Group			Comparison group			Between group difference; *P* value			
	N	Mean (SD)	95% CI	N	Mean (SD)	95% CI	Unadjusted mean (95% CI)	***p***-value	Adjusted for all SAP variables (95% CI)	***p***-value
Changes in physical activity measures										
Number of steps/day (Activpal)										
Baseline	59	9723 (3677)		66	9182 (3404)					
12 months	42	9444 (3800)		47	8548 (3160)					
Difference to baseline	36	−69.3(3019	−952 to 1091	44	−435 (2104)	−1074 to 205	689 (−257 to 1634)	0.153	483 (−635 to 1602)	0.393
Self-reported mins of physical activity/week ^a^										
Baseline	279	882 (783)		280	879 (676)					
12 weeks	262	964 (605)		264	921 (630)					
Difference to baseline	262	90 (679)	7 to 172	263	43 (598)	−30 to 115				
12 months	227	1046 (754)		239	906 (538)					
Difference to baseline	227	180 (617)	99 to 261	238	45 (660)	−39 to 129	1.1 (0.9 to 1.2)	0.316	1.1 (1.0 to 1.3)	0.123
Sitting Time (min)										
Baseline	59	1052.4 (97.5)		66	1048.4 (89.8)					
12 months	42	1050.9 (109.7)		47	1054.5 (97.5)					
Difference to baseline	36	0.1 (105.2)	−34.4. to 34.7	44	13.0 (81.5)	−11.8 to 34.7	0.8 (−25.5 to 27.1)	0.953	−12.9 (−52.6 to 26.9)	0.522

^a^log transformed

The secondary outcome of self-reported physical activity indicated that both groups increased activity over the study period with a moderately higher amount reported in the intervention group (mean difference ns). There was no difference in sedentary behaviour between groups. Following standard practice with self-reported physical activity we capped the maximum daily activity data at 360 mins/day [33] and when this was applied there was a significantly greater increase in activity in favour of the intervention group (Table 6).

**Table 6:** Changes in physical activity (curtailed to 360 mins/day)

Baseline and follow up measures										
	Intervention group			Comparison group			Between group difference; ***P*** value			
	N	Mean (SD)	95%	N	Mean (SD)	95%	Unadjusted mean (95% CI)	***p***-value	Adjusted for all SAP variables (95% CI)	***p***-value
Self-reported mins of physical activity/week (curtailed to 360 mins/day)										
Baseline	279	841(574)	773 to 908	280	856 (581)	787 to 924				
12 months	227	1009 (610)	929 to 1084	239	901(523)	835 to 968				
Difference to baseline	227	178.3	104.9 to 251.7	238	60.9	−12.3 to 134.1	39.4 (−31.2 to 109.9)	0.274	119.6 (17.1 to 222.0)	0.022

#### Health Behaviours and psychosocial variables

No significant differences between groups were detected in alcohol or fruit and vegetable intake (Table 7). It is notable that reported changes in attempts to alter physical activity and alcohol over the study period indicated a significantly greater odds of positive changes in the intervention group (Table 8). No significant differences were found for any of the psychosocial variables or EQ-5D quality of life domains (Table 9).

**Table 7:** Changes in key health behaviours

Baseline and follow up measures										
	Intervention Group			Comparison group			Between group difference; *P* value			
	N	Mean (SD)	95% CI	N	Mean (SD)	95% CI	Unadjusted mean (95% CI)	***p***-value	Adjusted for all SAP variables (95% CI)	***p***-value
Alcohol use (Audit-C score)^a^										
Baseline	279	4.5 (2.75)		281	4.2 (2.56)					
12 months	227	3.9 (2.57)		240	3.8 (2.50)					
Difference to baseline	227	−0.5 (1.55)	−0.74 to −0.34	240	−0.4 (1.59)	− 0.55 to − 0.15	0.22 (− 0.11 to 0.54)	0.197	0.13 (− 0.49 to 0.75)	0.681
Total fruit and vegetables (portions/day)										
Baseline	279	5.1 (2.11)		281	5.1 (2.44)					
12 weeks	262	6.0 (2.06)		264	5.8 (2.66)					
Difference to baseline	262	0.9 (1.77)	0.64 to 1.07	264	0.6 (2.01)					
12 months	227	6.1 (2.20)		240	5.8 (2.47)					
Difference to baseline	227	0.9 (1.85)	0.70 to 1.18	240	0.6 (1.90)	0.39 to 0.88	0.17 (−0.11 to 0.46)	0.234	0.30 (−0.04 to 0.64)	0.080

^a^Gamma distribution with identity link

**Table 8:** Changes in Reported lifestyle changes

Baseline and follow up measures										
	Intervention Group		Comparison group		Between group difference; ***P*** value					
	N	N (%)	N	Mean (SD)	OR Unadjusted (95% CI)	***p***-value	OR Adjusted for baseline (95% CI)	***p***-value	OR Adjusted for all SAP variables (95% CI)	***p***-value
**Attempted to lose weight**^**a**^										
**Baseline**	279	245 (88%)	281	265 (94%)						
**12 months**	227	102(45%)	240	165(69%)						
**Difference to baseline**					0.46 (0.34 to 0.62)	< 0.0001	0.40 (0.28 to 0.58)	0.667	0.98 (0.40 to 2.43)	0.618
**Attempted to increase physical activity**^**a**^										
**Baseline**	279	250 (90%)	281	255 (91%)						
**12 months**	227	212 (93%)	240	166(69%)						
**Difference to baseline**					2.49 (1.71 to 3.64)	< 0.0001	2.35 (1.57 to 3.53)	< 0.0001	2.48 (0.81 to 7.56)	< 0.0001
**Attempted to reduce alcohol intake**^**a**^										
**Baseline**	279	82 (29%)	281	89(32%)						
**12 months**	227	90(40%)	240	66 (28%)						
**Difference to baseline**					1.22 (0.94 to 1.58)	0.145	1.25 (0.96 to 1.63)	0.015	1.51 (0.78 to 2.94)	0.012

^**a**^ logistic regression on binary variables for SAP variables except number of visits (close co-linearity with intervention allocation)

**Table 9:** Changes in key quality of life outcomes

Baseline and follow up measures										
	Intervention Group			Comparison group			Between group difference; *P* value			
	N	Mean (SD)	95% CI	N	Mean (SD)	95% CI	Unadjusted mean (95% CI)	***p***-value	Adjusted for all SAP variables (95% CI)	***p***-value
EQ 5 D Health Index Score^a^										
Baseline	279	0.9 (0.14)		281	0.9 (0.15)					
12 weeks	262	0.9 (0.14)		264	0.9 (0.16)					
Difference to baseline	262	0.0 (0.13)	0.00 to 0.04	264	0.0 (0.15)	−0.01 to 0.03				
12 months	227	0.8 (0.18)		240	0.8 (0.17)					
Difference to baseline	227	−0.0 (0.15)	− 0.03 to 0.02	240	− 0.01 (0.14)	− 0.03 to 0.01	0.00 (− 0.02 to 0.02)	0.990	0.01 (− 0.04 to 0.05)	0.726
EQ 5 D Health state today										
Baseline	279	75.2 (16.75)		281	75.2 (15.41)					
12 weeks	262	80.9 (13.68)		264	80.2 (14.87)					
Difference to baseline	262	5.4 (13.81)	3.73 to 7.09	264	5.3 (13.16)	3.67 to 6.86				
12 months	227	80.7 (13.87)		240	78.5 (15.16)					
Difference to baseline	227	5.0 (15.71)	2.98 to 7.09	240	2.5 (14.14)	0.70 to 4.29	5.45 (1.81 to 9.10)	0.004	0.57 (−4.60 to 5.75)	0.827

^a^Gamma distribution with identity link

#### Cardiovascular and diabetes risk

No significant differences between groups were detected although both measures of insulin and total cholesterol indicate a favourable direction of change (Table 10).

**Table 10:** Changes in key cardiovascular measures

Baseline and follow up measures										
	Intervention Group			Comparison group			Between group difference; *P* value			
	N	Mean (SD)	95% CI	N	Mean (SD)	95% CI	Unadjusted mean (95% CI)	***P*** value	Adjusted for all SAP variables (95% CI)	***P*** value
HbA1C mmol/mol										
Baseline	256	39.11 (6.70)		272	38.88 (5.46)					
12 months	200	39.70 (7.37)		220	39.20 (6.32)					
Difference to baseline *transformed*	199	0.46 (3.77)	−0.07 to 0.98	220	0.19 (2.49)	−0.14 to 0.52	1.01 (0.99 to 1.02)	0.510	1.01 (1.0 to 1.02)	0.241
Insulin uU/ml										
Baseline	256	22.41 (29.42)		272	20.54 (25.56)					
12 months	203	17.16 (19.00)		221	20.89 (26.86)					
Difference to baseline *transformed*	202	−4.86 (23.95)	−8.18 to −1.53	221	0.36 (34.82)	−4.25 to 4.98	0.95 (0.85 to 1.07)	0.405	0.84 (0.71 to 1.01)	0.057
Total cholesterol mmol/L^a^										
Baseline	256	5.10 (0.98)		272	5.03 (0.90)					
12 months	203	5.04 (0.96)		221	5.07 (0.95)					
Difference to baseline *transformed*	202	−0.05 (0.74)	−0.16 to 0.05	221	0.09 (0.57)	0.01 to 0.16	1.00 (0.98 to 1.03)	0.752	0.98 (0.95 to 1.00)	0.072
HDL cholesterol mmol/L^a^										
Baseline	256	1.34 (0.31)		272	1.36 (0.34)					
12 months	203	1.35 (0.31)		221	1.39 (0.35)					
Difference to baseline *transformed*	202	0.01 (0.19)	−0.02 to 0.04	221	0.02 (0.17)	−0.00 to 0.04	0.98 (0.95 to 1.01)	0.267	0.99 (0.97 to 1.02)	0.541
Triglycerides mmol/L^a^										
Baseline	256	1.52 (0.70)		272	1.46 (0.75)					
12 months	203	1.47 (0.70)		221		1.39 to 1.61				
Difference to baseline *transformed*	202	−0.04 (0.66)	−0.13 to 0.06	221	0.03 (0.57)	−0.04 to 0.11	1.03 (0.98 to 1.09)	0.280	0.96 (0.90 to 1.03)	0.265
Mean Systolic blood pressure mmHg^a^										
Baseline	279	129.76 (16.22)		280	131.93 (16.18)					
12 months	226	129.35 (16.82)		238	131.29 (16.96)					
Difference to baseline *transformed*	226	−0.61 (13.06)	−2.32 to 1.10	237	−0.59 (14.87)	−2.50 to 1.31	0.98 (0.97 to 1.00)	0.039	1.00 (0.98 to 1.02)	0.976
Mean Diastolic blood pressure mmHg^a^										
Baseline	279	80.14 (10.45)		280	80.94 (10.33)					
12 months	226	79.74 (10.10)		238	80.13 (9.81)	78.87 to 81.38				
Difference to baseline *transformed*	226	−0.36 (8.14)	−1.42 to 0.71	237	−0.44 (8.78)	−1.57 to 0.68	0.99 (0.98 to 1.01)	0.318	1.00 (0.98 to 1.02)	0.888

^a^log transformed

#### Adverse events

No adverse events reported by participants were related to participation in the trial (Table 11).

**Table 11:** Serious Adverse Events Summary

	Intervention	Comparison	Total
All participants	279	281	560
Participants without serious adverse events	278	278	556
All adverse events	98	114	212
All serious adverse events	2	4	6
Cerebrovascular accident	0 (0.0%)	1 (0.9%)	1 (0.5%)
Coronary artery disease	1 (1.0%)	0 (0.0%)	1 (0.5%)
Fall	0 (0.0%)	1 (0.9%)	1 (0.5%)
Myocardial infarction	1 (1.0%)	0 (1.0%)	1 (0.5%)
Pulmonary embolism	0 (0.0%)	1 (0.9%)	1 (0.5%)
Urosepsis	0 (0.0%)	1 (0.0%)	1 (0.5%)

#### Health economic analysis

**Fig. 3:**
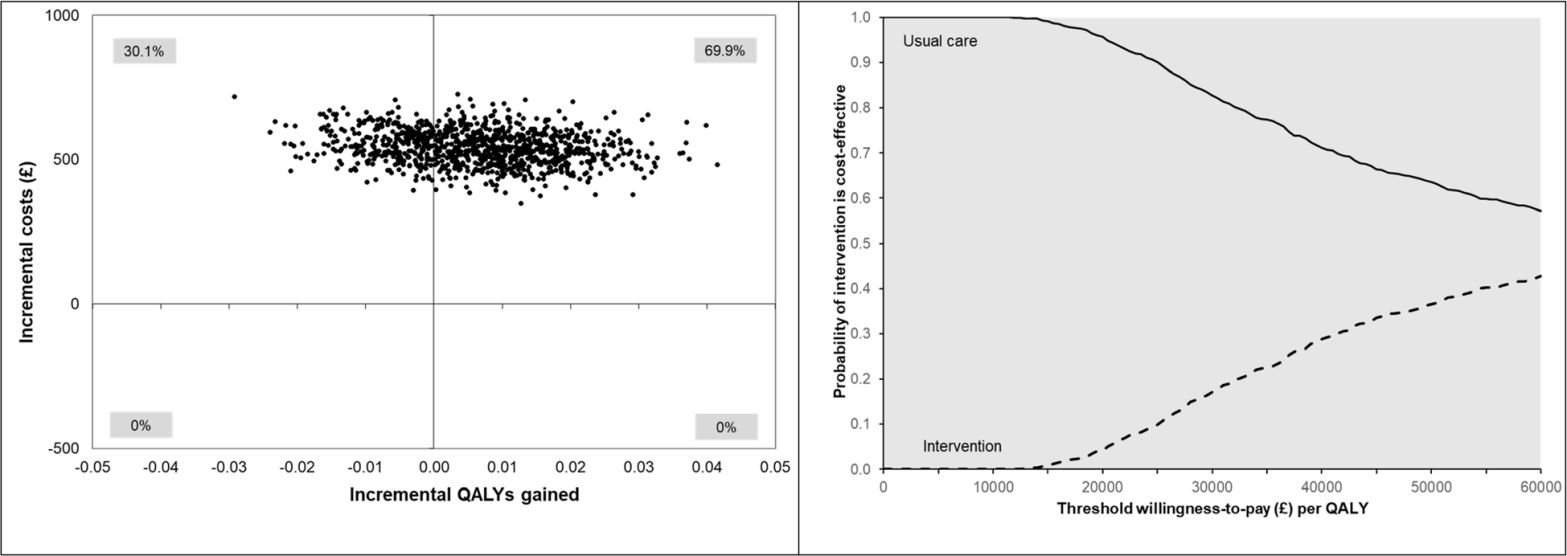
Probability of cost-effectiveness, Primary analysis over 12 months, using complete cases (*n* = 452)

Intervention costs are presented in Table 12. Table 13 indicates that the health care costs were significantly higher in the intervention group relative to the comparison group. There was a non-significant gain in EQ-5D in the intervention group relative to the comparison group. The incremental cost per QALY gained values ranged from £55,255 to £99,804 per QALY gained. Figure 3 indicates that the probability of the intervention being judged as cost-effective is below 20% (using the conventional threshold of cost-effectiveness as cost per QALY gain of up to £30,000 or less).

**Table 12:** Breakdown of intervention costs per participant

Category	Component	Primary analysis	SA #1	SA #2	SA #3
Staff time	ActWELL Project Officer Salary	67,373	56,144	78,602	37,430
	Office Co-ordinator (equivalent salary £22,000 pa)	5192	5192	5192	5192
	Management Cost	30,288	30,288	30,288	10,500
	Hays - Temp recruitment	8250	8250	8250	8250
	Communications officer time (equivalent salary £32, 500 pa) 16 days	2000	2000	2000	2000
Staff training	ActWELL Project Officer Training & Associated costs	1040	1040	1040	1040
Staff travel	ActWELL Project Officer travel costs for meetings related to ActWELL (TMG and leisure centres)	741	741	741	741
	ActWELL Project Officer Travel costs for volunteer support	1439	1439	1439	1439
Ad cost	Facebook paid for ads	250	250	250	250
Office supplies	ActWELL office supplies: Locking boxes for storing personal data, resources for events	189	189	189	189
	ActWELL Postage for ActWELL packs and returning mobile phones	181	181	181	–
Coach training	Venue	–	–	–	–
	Accommodation	2943	2943	2943	2943
	Catering	2815	2815	2815	2815
	Trainer:				
	*A*	2433	2433	2433	2433
	*B*	1265	1265	1265	1265
	*C*	999	999	999	999
	Coach pack	1642	1642	1642	1642
Delivery to participants	Mobile phone costs - coaches, equivalent annual cost at 3.5% per annum	1965	1965	1965	1965
	Travel expenses - coaches (intervention visits)	5631	5631	5631	5631
	Participant pack - production costs	4342	4342	4342	4342
Total cost	Sum of staff time, staff training, staff travel, ad cost, office supplies, coach training, delivery to participants	140,978	129,749	152,207	91,065
**Cost per participant (£)**	***N*** **= 279**	**505**	**465**	**546**	**326**

Primary analysis: 60% time spent by project officer on intervention, Jan 2017-Dec 2019

Sensitivity Analysis 1 (SA#1): 50% time spent by project officer on intervention, Jan 2017-Dec 2019

Sensitivity Analysis 2 (SA#2): 70% time spent by project officer on intervention, Jan 2017-Dec 2019

Sensitivity Analysis 3 (SA#3): Costs expected when ActWELL is rolled out and in steady state (assumes only annual salary for project officer and 18% on-costs)

**Table 13:** Adjusted^a^ mean incremental costs, incremental QALYs, and incremental cost-effectiveness ratio over 12 months between intervention group vs usual care group from NHS perspective

Analysis	Incremental mean costs, £ (95% CI)^b,c,d^	Incremental mean QALYs (95% CI)^b,c,d^	ICER (£/QALY)
Complete cases^e^	541.74 (429.71 to 656.68)	0.006 (−0.015 to 0.029)	83,440
SA: Decrease time spent to 50% by staff on intervention-related activities (complete case)	500.72 (388.85 to 615.17)	0.006 (−0.015 to 0.029)	77,123
SA: Increase time spent to 70% by staff on intervention-related activities (complete case)	582.68 (470.66 to 698.07)	0.006 (−0.015 to 0.029)	89,746
SA: Lower intervention cost (complete cases)^f^	358.74 (247.92 to 471.38)	0.006 (−0.015 to 0.029)	55,255
SA: Imputed dataset	548.94 (447.10 to 649.20)	0.006 (−0.012 to 0.022)	99,804

*Abbreviations*: *QALYs* quality-adjusted life-years, *ICER* incremental cost-effectiveness ratio, *SA* sensitivity analysis

^a^Adjusted for baseline differences (age, Scottish Index of Multiple Deprivation, employment status, smoking status, body mass index, alcohol intake, eating habits, physical activity time, baseline EQ-5D health utility score and baseline cost)

^b^Bootstrapped non-parametric 95% confidence interval (2.5th/97.5th percentile)

^c^Generalised linear model with Poisson distribution and power 0.5 link function to estimate incremental costs and generalised linear model with Gaussian distribution and identity link function to estimate incremental QALYs (complete cases)

^d^Discounted at 3.5% per year

^e^Included intervention cost of £505 per participant. This consists of staff cost, lifestyle coaches training cost and ActWELL delivery cost. Based on the activities performed from Jan 2017 to Dec 2019, 60% staff time was spent on coach recruitment, training, support and management

^f^Included intervention cost of £326 per participant. This considers costs that would accrue when ActWELL programme is rolled out in ‘real life’ and in steady state. Staff cost (1-year salary of all staffs), training cost and ActWELL delivery cost were included

#### Intervention invitation, delivery, acceptability and responses

The invitation to participate in the intervention was dependent on the introduction and endorsement by NHSSBS screening staff. Interviews with the four ActWELL mammography champions indicated that there had been some concerns prior to the start of recruitment: that there may not be time during the tight clinic schedule to answer questions raised by women about the study; that women might be upset by the mention of lifestyle issues, including weight, during the screening process; that mentioning the study during screening might impact negatively on women’s willingness to attend for screening in future. However, no mammographers reported any instances of women feeling distressed about raising the issue of lifestyle. The mammographers described how the ActWELL introduction process generally became embedded into practice, sometimes with modifications, although these were generally minor in nature. No mammographers felt that including the ActWELL introduction in the screening process had impacted negatively on women’s willingness to attend for screening (Table 14).

**Table 14:** Summary of Mammographers’ experience of the ActWELL study

**Initial responses to the study proposal**	• In terms of response to the proposal of involving mammographers in study interest, the response was mixed. In one service, the staff team were excited to be involved in the study because they perceived this as a valuable opportunity for women which in turn may have a positive impact on attendance rates. In contrast, in another service, (one that had previously been involved in a pilot of the intervention), the champion described a reluctance on the part of colleagues to become involved again. However, she also remarked that this may simply have represented resistance to another change to routines.
**Mammographers’ perceptions of the study purpose**	• ActWELL champions understood and appreciated that the purpose of the study was to reduce women’s risk of breast cancer by addressing lifestyle factors. As such there appeared to be no resistance from mammographers towards the study premise, yet one champion, had reservations about the way in which women were approached about the study, demonstrated concerns about discussing such an emotive topic as cancer risk and linking this with weight in what is a very brief clinical appointment.
**Embedding ActWELL introduction into practice**	• Mammographers recognised that the key to implementing the ActWELL recruitment task was to make it part of the clinic practice routine and described ways in which this was achieved.
	• Generally it was felt that, as the recruitment became embedded into practice, the impact on consultation times and overall smooth running of the service was manageable: *“once we established a pattern for it, it was actually more achievable than we initially thought”*
	• It was noted, however, that there was limited time to answer any questions which women might raise without impacting on the very tight appointment schedule. For one interviewee, the concern about lack of time was bound up with and reinforced the concerns expressed in another service about the inappropriateness of telling women about the link between unhealthy lifestyle and cancer risk.
**Barriers and challenges**	• Generally, these were practical, with time pressures being most consistently identified as an issue which affected both staff and the women themselves: *“it did feel a bit rushed, and it wasn’t fair on the ladies, but you can only do what you can with the time you are given”.*
**Perceptions of women’s response and information needs**	• Despite concerns expressed by one mammographer that women might feel it inappropriate to have the issue of lifestyle and cancer risk mentioned during mammography, no mammographers mentioned in the interviews any instances of women feeling distressed, although it should also be noted that the tight timing of appointments meant there was limited time for staff to gauge how women felt.
	• No mammographers felt that including the ActWELL recruitment in the screening process had impacted negatively on women’s willingness to attend for screening.
**Modifications**	• In terms of suggested modifications to the recruitment process itself, these were generally minor in nature.

Post study interviews with 24 ActWELL intervention and eight comparison participants indicated that the mammography setting was felt to have been an appropriate recruitment channel (Table 15). It is notable in the 12 months follow up exit questionnaires that 90.4% of 167 intervention participants who completed follow up measures reported they would still have been interested in the opportunity for a lifestyle intervention if this had not been a research study.

**Table 15:** Summary of Participants’ views on the Actwell study (procedures and intervention)

24 participants were interviewed by telephone using a semi-structured interview guide. The sample was selected to represent all study areas and a range of socio-economic backgrounds using SIMD	
**Views on the study and intervention**	
**Recruitment**	• Most women recalled finding out about the study through ‘leaflets’, ‘cards’ or ‘posters’ at the mammography clinic – only a minority recalled any conversation about it. • Study information materials were felt to have been clear and helpful. The only area of uncertainty was around the term ‘lifestyle coach’, which conjured up expectations for some of a more personalised, intensive form of support. • The mammography setting was felt to have been an appropriate recruitment channel
**Motives for participation**	• Motives for participation were both altruistic (to support worthwhile research, to help find out about preventing cancer) and self-help/improvement (particularly, to lose weight, and generally to improve health). Sometimes both types of motive were present, reinforcing each other. For some, Actwell had come along at a key moment (change of routine, big birthday, awareness of own mortality, family illness). • Breast cancer prevention was not necessarily a salient factor for many, and there was low awareness of BCN involvement.
**Acceptability and convenience**	• Research nurse appointments were felt to have been pleasant and well handled. • Venues for lifestyle coach meetings were mostly felt to have been appropriate, although some had found them difficult to get to, and the rooms available had not always been very pleasant. • Telephone calls were mostly felt to have been acceptable and convenient, and of appropriate frequency and duration. • Views on overall mix and timing of face-to-face meetings and telephone calls: mostly, participants felt this was about right, although some felt they would have welcomed more face-to-face support.
**Views on the lifestyle coaches**	• Generally coaches were highly regarded. Seen as pleasant, warm, positive, although one participant reacted negatively. • Usually coaches were perceived as empathetic, understanding, and non-judgemental, although some felt that ‘slim’ coaches did not necessarily understand the challenges faced by overweight women. • Generally praised for quality of support provided, with some coaches being described as having particular insight and skill in knowing how to motivate change. • Some interviewees had expected that coaches would have a background in lifestyle coaching or specialist knowledge (for example, concerning particular conditions and dietary needs). Some participants had not been aware at start that all coaches would be volunteers.
**Views on the intervention**	• Goal setting appeared generally to have worked well, with participants feeling they had been appropriately involved, and goals being perceived as realistic and manageable. • Varying views on the information and advice provided. In some cases, seen as not specific enough, or not telling participants anything new. • For some participants, the move to telephone calls was disappointing as they lacked the rapport and accountability associated with face-to-face contact. Others, however, felt the phone calls provided sufficient support and encouragement. • Participants generally appreciated using the pedometers (although they were difficult to wear, compared with fitbit-type watches). There were more mixed views on regularly weighing themselves, with some finding it helpful and others demotivating.
**Suggestions for changes and improvements to Actwell**	Many felt ‘nothing’ needed changing, but some suggestions were offered: • around a third would have welcomed more contact with lifestyle coach, either during or after the 12 month period. • some suggested contact with other participants – ‘buddy’ system or an informal social group. • some would have liked feedback on the blood tests at baseline and follow-up
**Barriers and facilitators to change (analysed in relation to the COM-B model)**	
**Capability**	• Health (conditions which affected mobility, recent surgery), life events such as Christmas and holidays, and stressful periods, could reduce capability and make participants fall back into old patterns of treats and comfort eating.
**Opportunity**	• Work (for those still in employment), family caring commitments and looking after pets could reduce time available for activity and affect energy and motivation, but could also present opportunities for exercise. • Weather/lack of daylight and cost of accessing healthy food and leisure facilities were negative factors for some. Several commented on free activity options such as walking on beach.
**Motivation**	• Could be both a positive and negative factor; some were strongly driven and self-motivated, others needed external boosts to motivation such as the regular contact with the coach. Personal goals, such as being fitter to play with grandchildren, were helpful. • Mixed experiences of family and friend support.

Post study interviews with eight coaches (Table 16) indicated that the intervention organisation process was well managed and straightforward. Overall, coaches felt a high level of confidence in carrying out their role, although some indicated nervousness and uncertainty in the initial stages and benefited from opportunities to practise and gain experience. Coaches noted a generally good level of engagement among ActWELL participants, with most starting off very enthusiastic. Over time, coaches found that engagement amongst participants varied, particularly after the face-to-face appointments ended and the intervention moved on to telephone support. One coach commented that most of their participants reported having lost a lot of weight before they had started on ActWELL, which may have impacted on further weight loss during the intervention.

**Table 16:** Summary of coaches’ views on the Actwell training and intervention

Eight coaches who met the following eligibility criteria undertook semi-structured interviews: • had seen a minimum of three intervention participants • represented the four areas participating in the ActWELL study • represented the different waves of ActWELL training (four coaches from the first wave of training, two from each of the second and third waves of training).	
**Background**	• The coaches had a range of backgrounds and experiences, including nursing, general practice, fitness coaching, voluntary work, education and nutrition. Some were retired while others were in work, sometimes also juggling other family commitments. Some had prior experience of breast cancer, either themselves or among their family and friends.
**Perceptions of study and role**	• The coaches clearly understood the prevention concept at the heart of ActWELL, and perceived that the aim of the study was to assess whether a lifestyle coach approach could encourage lifestyle changes, specifically relating to physical activity, diet, alcohol use and weight.
	• Lifestyle coaches understood that the nature of their role was primarily *“support and encouragement”* for women to identify changes they could make for themselves, rather than to direct them to follow a particular plan of action.
**Training**	• Lifestyle coaches generally enjoyed and appreciated the training. For some with prior experience of this kind of work it was felt to be sufficient and appropriate. However, others noted the training to be intense and hurried for the amount of learning required. The use of role play elicited mixed responses.
**Management of intervention procedures**	• All coaches praised the support they had received from Breast Cancer Now (BCN). The manager in charge of ActWELL volunteers was described as helpful, supportive, quick to respond and efficient.
	• The process was generally described as well managed and straightforward, and coaches particularly appreciated that there was flexibility to accommodate their particular requirements and circumstances. The types of queries which lifestyle coaches received typically concerned health problems experienced by intervention participants and the implications of these for their involvement in the study. Generally, lifestyle coaches found the process of scheduling appointments to be manageable and not too onerous.
	• Some had found the paperwork (including record forms for intervention and research purposes) which they had to complete after each session and telephone call manageable and straightforward but other struggled.
	• The process of arranging meeting venues in local leisure centres was mostly straightforward, with leisure centre staff generally being described as helpful and accommodating, albeit centralised booking systems sometimes made it difficult to speak directly to the venue. However, some issues were experienced regarding room availability and suitability, particularly where the only space available was in a public area.
	• The process of scheduling and making telephone calls was generally experienced as unproblematic.
	• A consistent theme across the interviews was the duration of the face to face appointments, with coaches finding that the appointments, particularly the first one, could take much longer than had been recommended in the training. This was for two main reasons. Firstly, coaches found that it was important to build a rapport with the participant, and this took time. Secondly, the requirement in the first face to face appointment to take the participant for a 10 min walk ate substantially more into the appointment time than anticipated.
	• Lifestyle coaches were very positive about their experience of volunteering on the ActWELL programme, including speaking of their enjoyment of being in contact with and supporting participants.
	• In terms of their routines, coaches were generally able to accommodate their ActWELL involvement, although it was acknowledged that the time commitment was substantial and in some cases had exceeded initial expectations. However, generally, lifestyle coaches noted that it was made clear to them that volunteering to be a coach would be a substantial time commitment, and those who were interviewed took this commitment seriously.
**Participant interactions**	• Overall, coaches felt a high level of confidence in carrying out their role, although some indicated nervousness and uncertainty in the initial stages. Opportunities to practise and gain experience were helpful for those who started with lower confidence but saw this grow over time.
	• Coaches noted a generally good level of engagement in ActWELL participants, with most starting off very enthusiastic. Over time, coaches found that engagement in participants varied, particularly after the face to face appointments came to an end and the intervention moved on to telephone support. In some cases the process had been straightforward, with participants continuing to respond well to the programme in this second phase; however, in other cases the transition to telephone calls had been somewhat unsatisfactory.
	• Another coach commented that most of their participants had lost a lot of weight before they had started on ActWELL, and so few lost further weight during the intervention.
	• Multiple barriers and facilitators to participants’ progress within ActWELL were identified by coaches. Barriers reflected characteristics of participants’ lifestyles and routines, including the role of food in their lives; as well as their approach to, and understanding of ActWELL. Facilitators to progress were identified within the ActWELL programme and in participants themselves including their levels of self-motivation, and interest in physical activity, healthier diets and good health generally.
	• Coaches had various suggestions for improving ActWELL and these focussed on the format and content of the programme and better supporting coaches to prepare for and deliver the intervention.

Most (90%) participants attended both face-to-face consultations (mean visits 1.9 SD 0.28) and 59% completed the 9 planned telephone calls (mean 7.1 SD 2.81). Self-reported fidelity data was provided by 32 coaches who described “always” delivering key intervention components (*n* = 7) (range 67 to 96%). Independent fidelity analysis of recordings of 35 coaching sessions and 22 support calls found 69–88% adherence to protocol.

Data from the study exit questionnaires (*n* = 167) showed that most (91%) intervention participants said they found the face-to face contact very (73%) or quite (18%) helpful. Telephone contact was also rated highly with most participants reporting that they found it very (61%) or quite (23%) helpful. Overall, participants rated the programme well and 90% said they were very (63%) or quite (27%) likely to recommend it to others.

Post study interviews (*n* = 24) with intervention participants [Flow Diagram B] indicated that coaches were generally highly regarded (Fig. 2). Four main areas were highlighted: the coach’s personality or manner, ability to empathise, the support provided throughout the programme, and their ability to understand how to motivate change. Other intervention aspects which were highlighted related to behavioural change techniques utilised (goal setting, telephone support, self-monitoring of step counts). [Flow Diagram B] (Fig. 2).

In terms of changes in lifestyle, interviewees tended to report a number of small rather than major changes to their diet. Participants spoke of increasing their fruit and vegetable intake, replacing foods and drinks, and greater portion control. There was some evidence from the interviews that participants had high levels of activity prior to ActWELL. However, one participant said that she felt her baseline number of steps was artificially high as she had increased her activity prior to meeting the lifestyle coach.

## Discussion

The results indicate that breast screening provides a promising and acceptable opportunity to initiate a weight management intervention programme. The delivery of the programme by volunteer coaches in leisure centres was achieved to a high degree of fidelity and resulted in clinically relevant weight loss, doubling the likelihood of achieving weight loss (5%) at 12 months with potential to decrease breast cancer risk. The weight loss is relatively modest but in line with that achieved by Ayeyard et al [34] (1·43 kg (95% CI 0·89–1·97) where weight loss was initiated in a primary care setting with the potential for significant impact at a population wide level if scaled up accordingly.

The programme design used an innovative approach for the engagement and delivery of a weight management programme, which has the potential to be rolled out across screening communities. This study adds trained volunteers to the groups (including veterans [35] and peer workers [36, 37]) who have been shown to successfully enhance existing efforts in obesity control. The demand and uptake of the programme was higher than anticipated and considerably higher than the number returned in the feasibility study. Qualitative data support the use of the screening setting as a “teachable moment” [38] and a trigger for considering health behaviours. Recent data from an Australian study [39] reported that 76.4% of 204,429 women agreed to have their height and weight measured at routine screening clinics, supporting the potential for initiating weight management advice/referrals.

The current programme provided an opportunity to engage with women from a wide range of sociodemographic backgrounds. The uptake across SIMD groups reflected the incidence distribution of breast cancer, the areas where the breast-screening programme was inviting women for mammography during the trial period, and interest in the programme. However, the uptake by women living in the most deprived areas was lower than that attained in the feasibility work, and ways to improve engagement deserve further exploration.

The response to the programme indicates that the approach taken was acceptable to women in the overweight and obese categories. The weight change detected was most likely due to small decreases in caloric intake by changes in a number of habitual behaviours (including portion sizes) rather than any one category of foods (e.g. sweet snacks, alcohol). Overall, the objective and subjective measures of physical activity suggest that participants were a very active group [40], although activity levels can often be inflated during periods of measurement and it is notable that all had excess body weight (which can make it harder to exercise). Responses to the query at *baseline* on whether participants had increased their physical activity (no time period indicated, but likely to include the time between recruitment and research nurse visit) show that 90% of participants said they had attempted to increase physical activity. These results suggest that the eligibility process could usefully have included a physical activity assessment. The impact of the intervention on physical activity is unclear because although the required number of participants received the accelerometers, too few were able to provide useable data.

The ActWELL intervention costs around £500 per participant (Table 13), which is comparable to a community-based weight management program run through the Scottish colorectal screening service (£546) [39] but more expensive than the only other widescale community based weight RCT reported in Scotland run through Scottish football clubs (£165) [41] or international football clubs (approx. £250) [42]. In terms of cost-effectiveness, the health economics analysis results indicate that the gains in health-related quality of life over 12 months are not sufficiently large enough to conclude that the intervention is likely to be judged as cost-effective. However, the impact of the intervention on long term health gain (i.e. reduction in breast cancer risk) cannot be assessed in this 12 month assessment. Itis possible that adopting a longer time perspective, by extrapolating the changes in weight observed within the study over a future period, would produce more favourable results [43]. Long-term modelling data [44] suggest that an intervention costing £500 used by overweight women aged 50–59 that led to a BMI reduction of 0.6 (comparable to ActWELL), with no regain of the weight lost, would have an incremental cost per QALY gained of under £10,000. Further longer-term follow-up of weight amongst study participants or additional modelling of impacts using our trial data would be required to test this hypothesis further.

Over 70% of Scottish women aged 50 to 74 years have excess body weight and 38% fail to meet physical activity recommendations [45] and are likely to benefit from lifestyle programmes that improve caloric intake and physical activity. In the current study, recruitment at BMI > 25 kg/m2 is important because many women will gain weight at this life stage, which is a risk factor for breast cancer (independent of actual BMI). Additionally, lifestyle habits may be worsening during the current COVID-19 pandemic. The current work demonstrates that partnerships between existing NHS services, leisure facilities and an enhanced role for third sector volunteers offer significant potential for greater ‘reach’ of weight loss programmes. Lifestyle interventions initiated in the breast cancer screening setting are a largely unexplored area [46], although repeated triennial appointments offer unique opportunities for initiation and re-enforcement of current evidence on lifestyle and breast cancer risk reduction and help to avoid a “certificate of health” effect [47]^.^ In 2007, Fisher et al. [48] reported that most women attending breast screening clinics were interested in receiving lifestyle advice, and an updated paper [49] reporting the views of 1803 women shows overwhelming support for receiving interventions.. Sinclair et al. [50] have also demonstrated that the breast clinic setting is an acceptable opportunity to discuss alcohol use. Using data obtained from a cross sectional population representative survey on early detection and prevention, Stevens et al. [50, 51] reported that across breast, cervical and colorectal screening programmes respondents report acceptability of and considerable willingness [51, 52] to receive healthy lifestyle advice and particularly weight management guidance in women with excess body weight. Health behaviour interventions offered within colorectal cancer screening programmes have also been shown to provide an effective route for change in lifestyle [41, 53].

However, future research is needed to identify how to achieve wider engagement with women living in more deprived circumstances and the challenge of achieving large weight loss and weight loss maintenance.

## Conclusions

A community weight management intervention initiated at breast screening clinics and delivered by volunteer coaches highlights significant opportunities for the use of a community assets approach to attain significant weight loss over a 12 month period and support breast cancer risk reduction in older women.

## Supplementary Information

**Additional file 1.**

**Additional file 2.**

## Data Availability

The authors are prepared to share anonymised participant data following reasonable request for same.
