# Correction to: A novel approach to increasing community capacity for weight management a volunteer-delivered programme (ActWELL) initiated within breast screening clinics: a randomised controlled trial

**DOI:** 10.1186/s12966-021-01232-6

**Published:** 2022-01-11

**Authors:** Annie S. Anderson, Huey Yi Chong, Angela M. Craigie, Peter T. Donnan, Stephanie Gallant, Amy Hickman, Chloe McAdam, Jennifer McKell, Paul McNamee, E. Jane Macaskill, Nanette Mutrie, Ronan E. O’Carroll, Petra Rauchhaus, Naveed Sattar, Martine Stead, Shaun Treweek

**Affiliations:** 1 Centre for Research into Cancer Prevention and Screening, University of Dundee, Ninewells Hospital & Medical School, Dundee, DD1 9SY UK; 2 Health Economics Research Unit, Institute of Applied Health Sciences, University of Aberdeen, Aberdeen, AB25 2ZD UK; 3 Division of Population Health and Genomics, University of Dundee, Ninewells Hospital & Medical School, Dundee, DD1 9SY UK; 4 Breast Cancer Now, 222 Leith Walk, Edinburgh, EH6 5EQ UK; 5 Physical Activity for Health Research Centre, University of Edinburgh, Saint Leonard’s Land, Holyrood Rd, Edinburgh, EH8 8AQ UK; 6 Institute for Social Marketing and Health, Faculty of Health Sciences and Sport, University of Stirling, Stirling, FK9 4LA UK; 7 Department of Breast Surgery, Level 6, Ninewells Hospital and Medical School, Dundee, DD1 9SY UK; 8 University of Stirling, Stirling, FK9 4LA UK; 9 Tayside Clinical Trials Unit, Tayside Medical Science Centre, Ninewells Hospital and Medical School, Dundee, DD1 9SY UK; 10 University of Glasgow, Institute of Cardiovascular and Medical Sciences, BHF Glasgow Cardiovascular Research Centre, 126 University Place, Glasgow, G12 8TA UK; 11 Health Services Research Unit, University of Aberdeen, Room 306, 3rd Floor, Health Sciences Building, Foresterhill, Aberdeen, AB25 2ZD UK

**Correction to: Int J Behav Nutr Phys Act 18, 34 (2021)**

**https://doi.org/10.1186/s12966-021-01099-7**

Following the publication of the original article [1], the authors identified that Fig. 1 was omitted. The figure is given below.

**Fig. 1:**
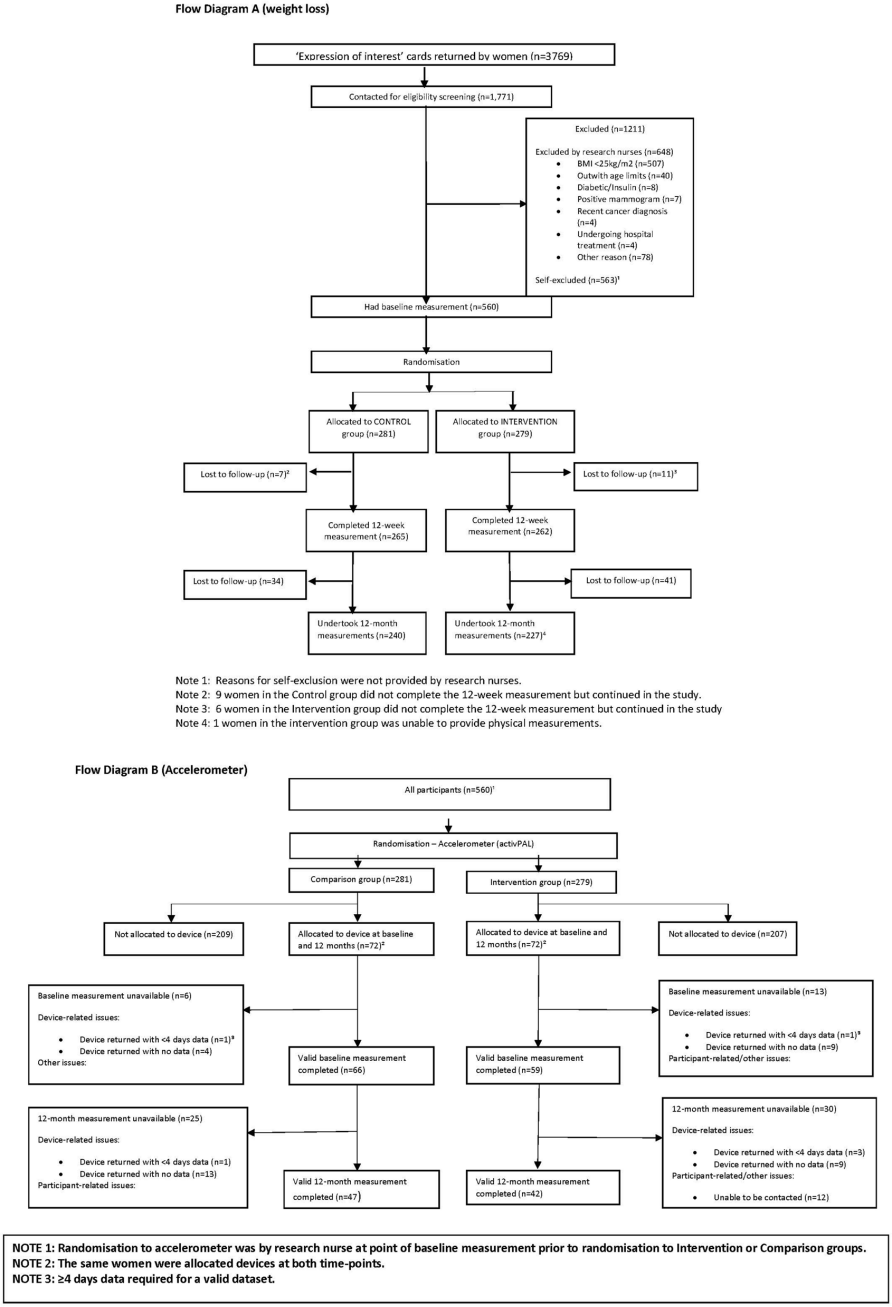
Probability of cost-effectiveness, Primary analysis over 12 months, using complete cases (*n* = 452)

The original article [1] has been corrected.
