# Supplementary material for: A novel approach to increasing community capacity for weight management a volunteer-delivered programme (ActWELL) initiated within breast screening clinics: a randomised controlled trial
Source: Int J Behav Nutr Phys Act. 2021 Mar 6;18:34. doi: 10.1186/s12966-021-01099-7 (PMC7936444; doi:10.1186/s12966-021-01099-7)
Supplement: Supplementary file 2 — Additional file 2. [file 12966_2021_1099_MOESM2_ESM.docx]

**
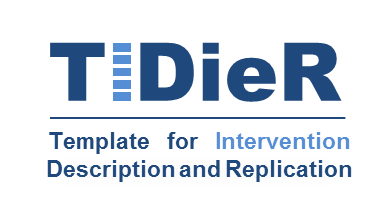
The TIDieR (Template for Intervention Description and Replication) Checklist*:**

1. **Brief Name**

ActWELL

1. **WHY**

Following the COM-B model the intervention aims to incorporate increased **M**otivation for weight loss (through awareness raising within the teachable moment setting) combined with increased **C**apability for effective lifestyle change combined with **O**pportunities for greater physical activity through an emphasis on walking initiatives and other community facilities using taxonomy-derived effective behaviour change techniques.

1. **WHAT**
2. **Materials**

**Visit 1**

- Touch, Look, Check leaflet (<http://breastcancernow.org/sites/default/files/public/tlc_breast_awareness_guide.pdf>)
- Breast cancer and lifestyle FAQ and links to science base (WCRF) (in house developed)
- BMI chart to identify BMI number (NHS)
- Assessing baseline step count (in house developed)
- Physical activity recommendations from UK Department of Health
- Kettlecise ways to reduce sedentary behaviour (<http://www.cancerpreventionscotland.org.uk/resources/kettlecise/>)
- Warm up exercises (in house developed)
- Walking Plan and Diary (in house developed)
- Implementation intention sheets (in house developed)
- Information sheet on calories and alcohol infographic <https://www.behance.net/gallery/5830267/Calories-Alcohol-infographic>
- Sugar savvy quiz https://www.wcrf-uk.org/sites/default/files/are-you-sugar-savvy-game.pdf
- Weight awareness plan (weekly weight logbook) (in house developed)
- Five day food and drink diary (in house developed)
- EatWELL Guide (NHS Tayside)
- Personalised weight loss plan (British Heart Foundation )
- Implementation Intentions sheet (in house developed)
- Food Labelling Guidance – credit card size (NHS Tayside)
- British Heart Foundation Booklet - Your simple guide to weight loss
- https://www.bhf.org.uk/publications/healthy-eating-and-drinking/facts-not-fads---your-simple-guide-to-healthy-weight-loss

1. **Procedures**

Overall: Two face to face visits plus 9 monthly phone contacts

**Visit 1 (60 minutes)**

- Self-identification of BMI

- Instruct participant on pedometer use and proposed walking programme
- Walk and talk 10 min

(Interactive walking session and discussion about increasing physical activity)

- Physical activity goal setting

(Implementation intention setting and personalised walking programme)

- Discuss how to reduce sedentary behaviour
- Caloric value of (hot and cold) alcohol and sugary drinks discussed

“Sugar Savvy” quiz undertaken (https://www.wcrf-uk.org/sites/default/files/are-you-sugar-savvy-game.pdf)

(Advice given on alternatives, portion size, frequency)

(Possibility of implementation intention setting on drinks)

- Weight loss goal

(Emphasis on modest up to 7% in 12 months)

- Motivational interviewing questions on weight loss
- Guidance on weekly self-weighing, reporting and feedback–

(Implementation intention setting for weighing– scales available for home use)

- Initial dietary challenges – snacking and “weakness foods”

(Based on a verbal 24 hour intake)

- Summarise meeting – goals set, times of relapse
  - All participants invited to undertake a 24 hour written diet recall for 5 days for review at visit 2

**Visit 2 (45 minutes)**

- Praise success (however modest)
- Evaluate and appraise PA goals

(Discuss how they feel physically and mentally about success of walking, or problems and possible solutions, review goals)

- Check body weight recorded
- Reminder about body weight and breast cancer risk reduction (even after 50)
- Highlight weight loss principles

(revising snacking, importance of meal patterns and 5 a day)

- Remind about goal set for weight loss and how this converts to personal eating plan
- Review 24 hour diet recall sheets (handed out last visit)

(or take a 24 hour recall if sheets not completed)

- Discuss calories – focus on -600kcal deficit diet

(Identify personalised eating plan using British Heart Foundation (BHF) materials)

- Discuss Portion sizes and frequencies

(use images from BHF materials and portion distortion information)

- Food labelling

(identify energy values for low and high, advise avoiding red TL, advise using at home)

- Identify implementation intentions on one food/drinking habit

(set one only- if suggestions are needed base on 24 hour recordings)

- - Summarise goals and key challenges, check all materials provided
  - Arrange first two telephone appointments
  - Discuss leisure centre activity to meet staff (if interested)

**Phone Calls**

- Check well being
- Check goal progress and self-reported weight
- Identify success and challenges
- Discuss possible problems ahead (e.g. holidays) and coping strategies

1. **WHO PROVIDED**

The intervention will be delivered by Breast Cancer Now volunteer coaches (http://breastcancernow.org). They will have a background and experience of counselling and receive a bespoke training programme by the research team including:

- Evidence base for reducing risks of breast cancer by lifestyle
- Key principles and application of healthy food and drinks choices, appropriate portions and coping with social consumption challenges
- Key principles and application of increased physical activity and reduced sedentary behaviour
- Key principles and application of weight management
- Personalisation of advice
- Motivational interviewing techniques (key questions only)
- Use of evidence-based behavioural change techniques (BCTs)
- Handling confidential data
- The intervention protocol and importance of its delivery. Confidentiality.
- Role play and assessments (face to face and telephone contact)

The training programme is likely to be delivered over 4 bespoke sessions. An exit certificate will be provided for those who successfully achieve the assessments and role play.

Role play and observations will be undertaken prior to commencing actual intervention.

1. **HOW**

Two individual, 1:1 coach to participant, face to face visits are planned (approx. 6 weeks apart) with monthly telephone contacts thereafter.

1. **WHERE**

The face to face visits are scheduled to take part in office space in local leisure centres. Each town has identified more than one possible venue and these will be noted. No home visits are scheduled and other locations are discouraged.

1. **WHEN and HOW MUCH**

The first face-to-face visit will take place within around 1 month of initial contact by the woman with the ActWELL team; the second will take place around 6 weeks later. The coaches will collect data for each participant on: date of contact, duration of contact and perceived engagement for both face to face and 9 telephone contacts. Other contacts e.g. SMS/email will also be noted. These data will allow dose, duration and outcomes to be assessed by individual coaches as well as overall.

1. **TAILORING**

Personalised advice is a key component of all aspects of the intervention. Motivational interviewing regarding weight loss will be undertaken to identify participant ambivalence and perceived personal advantages to weight management. In terms of **caloric prescription** this will be based on -600kcals required for weight loss (calculated using the equations from Miflin St. Jeor according to gender, age and body weight). Participant agreed goals (and implementation intentions) will be used for weighing and recording body weight (self-monitoring) and personalised feedback provided.

**Food and drink choices** will be based on information obtained from current eating habits obtained through 24 hour recalls to guide personalised advice on food frequency, portion sizes and foods to limit. Participant agreed goals (and implementation intentions) will be used for one specific food or drinking habit and this will be self monitored with personalised feedback provided.

**Physical activity dose** and duration will be based on the brief Scottish Physical Activity questionnaire to guide walking plans and sign post other activities (including those offered in the local leisure centres). Participant agreed goals (and implementation intentions) will be used for one specific aspect of habitual walking and this will be self-monitored with personalised feedback provided.

1. **MODIFICATIONS**

**Recommendations for modifications may arise from**

1. Feedback from coaches (individual issues or during regular round table meetings) or b) changes in evidence base for guidance or c) adverse events
2. **HOW WELL**

**Fidelity Procedures** We will undertake qualitative process measures to assess fidelity to the intervention. Time for implementation procedures will be recorded by intervention staff. Fidelity of programme delivery and content will be assessed by audio-recording and transcription of a random sample of LC face to face interactions and telephone contacts at each site. These will be compared to the protocol specified number of points to be covered in each session by a researcher at University of Stirling independent from the intervention.
